# Supplementary material for: Arbitrary aperture synthesis with nonlocal leaky-wave metasurface antennas
Source: Nat Commun. 2023 Jul 20;14:4380. doi: 10.1038/s41467-023-39818-2 (PMC10359259; doi:10.1038/s41467-023-39818-2)
Supplement: Supplementary file 1 — Supplementary Information [file 41467_2023_39818_MOESM1_ESM.pdf]

Supplemental Material for "Arbitrary Aperture Synthesis with Nonlocal Leaky-Wave Metasurface Antennas"

Gengyu Xu[1], Adam Overvig[1], Yoshiaki Kasahara[1,2], Enrica Martini[3], Stefano Maci[3] and Andrea Alù[1,4,5]✉

[1] Photonics Initiative, Advanced Science Research Center, City University of New York; New York, NY 10031, United States
[2] Department of Electrical Engineering, The University of Texas at Austin; Austin, TX 78712, United States
[3] Department of Information Engineering and Mathematics, University of Siena; Siena, 53100, Italy
[4] Department of Electrical Engineering, The City College of New York; New York, NY 10031, United States
[5] Physics Program, Graduate Center, City University of New York; New York, NY 10016, United States

✉ email: aalu@gc.cuny.edu

Supplementary Material

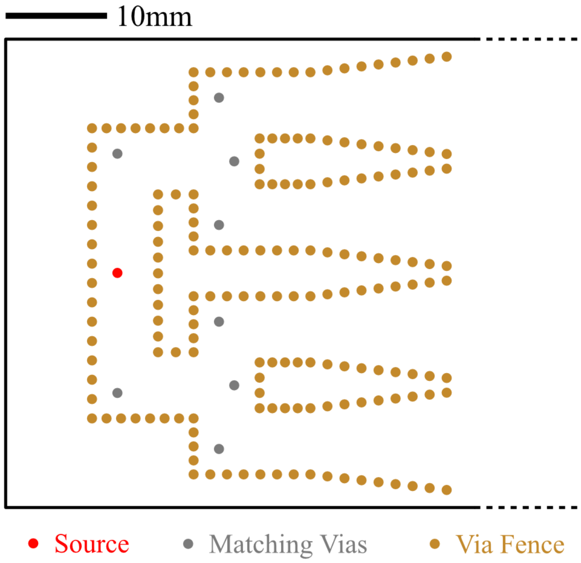

Supplementary Figure S1: Layout of the substrate integrated waveguide launcher employed in the LWM prototypes. The vias have 0.5mm radii and 1.9mm maximum pitch, which is smaller than 1/4 of the wavelength inside the dielectric substrate (at a frequency of 20.55GHz). The locations of the matching vias and the source have been optimized to maximize the bandwidth of the launcher (in the absence of the LWM).

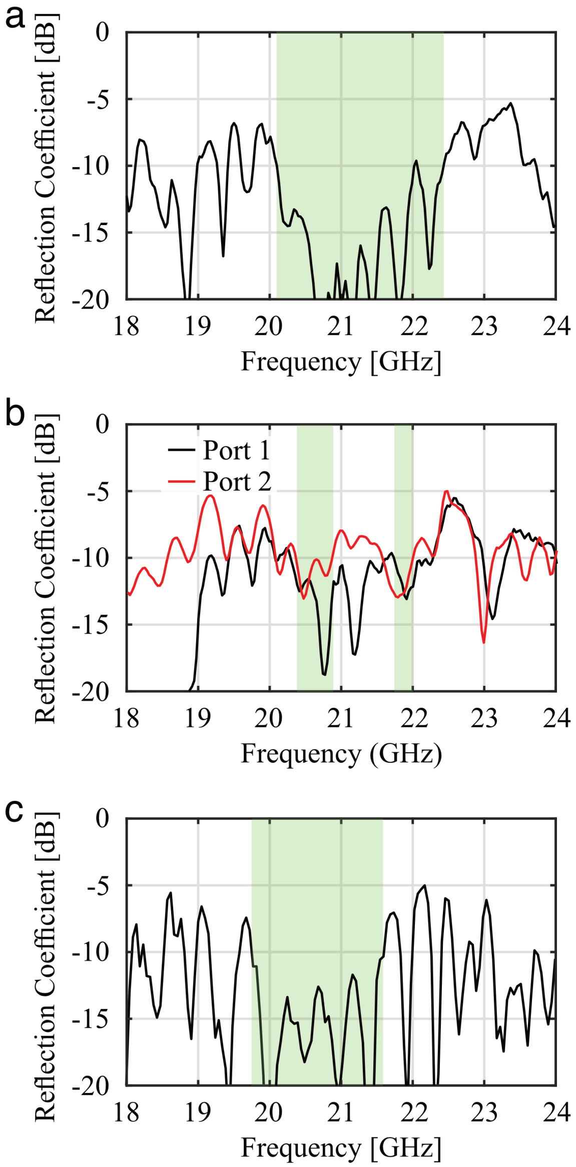

Supplementary Figure S2: Measured input reflection coefficients of the LWM prototypes. Results for (**a**) the SIMO LWM, (**b**) MIMO LWM, and the (**c**) multi-beam LWM antenna. The SIMO and multi-beam LWM exhibited around 10% -10dB fractional bandwidth (marked by the green shaded region). In comparison, the MIMO prototype had slightly reduced bandwidth due to the increased (but still acceptable) reflection at port 2.

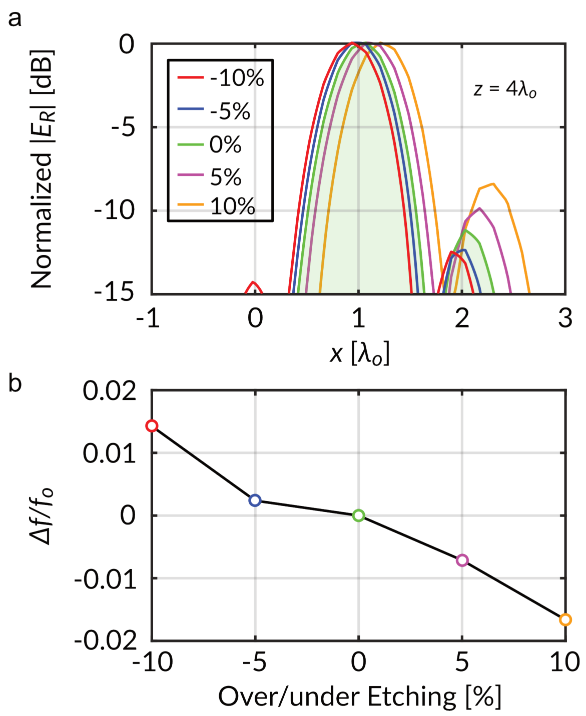

Supplementary Figure S3: Study on the robustness of the LWM functionality to etching tolerances during fabrication. As an illustrative example, the SIMO LWM prototype examined in the main text is studied here. The normalized magnitude of the RCP component in the designated RCP focal plane $z = 4\lambda_o$ is plotted in panel (**a**) for various degree of over- and under-etching. The percentage value indicates the size deviation of the open slots from their intended dimensions. The focal spots drift slightly from the intended position as we increase the amount of etching error, which can be compensated by slightly detuning the operating frequency. In panel (**b**), we plot the frequency deviation required to maintain the focal spot at its intended location ($x = \lambda_o$), for various degree of over- and under-etching. It is evident that $\pm 10\%$ etching error will shift the optimal operating frequency only by approximately 1.5%, meaning the proposed LWM is highly robust against this type of manufacturing defect (which is the most common in practice).

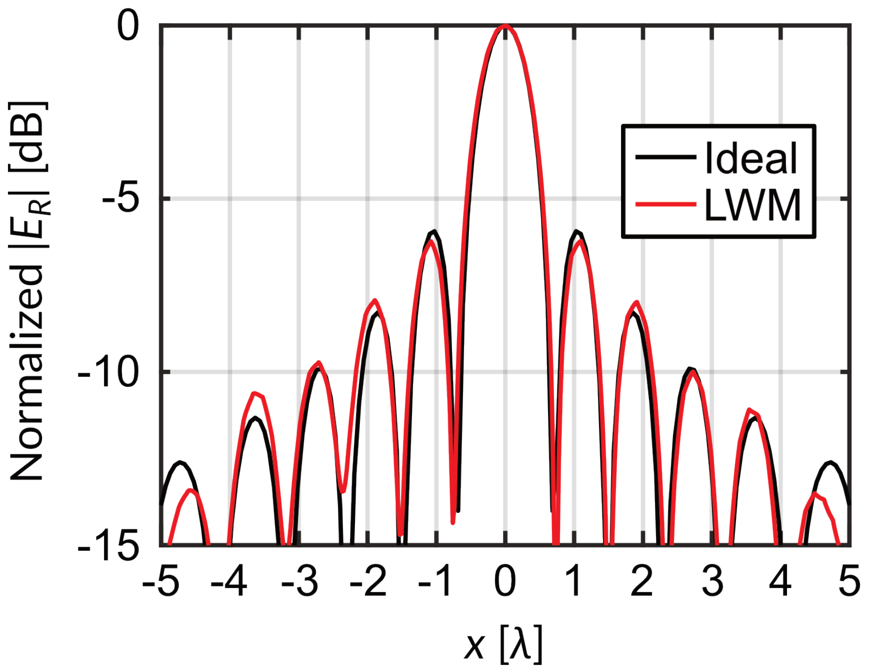

Supplementary Figure S4: Comparison of the focusing resolution of a typical LWM design with theoretical expectations from a tapered aperture. The red curve depicts the electric field intensity in the focal plane ($NA = 0.78$, $f = 20.55$ GHz) predicted by theory (Fourier optics), assuming a continuous aperture field distribution given by equation (5b) in the main text. The black curve corresponds to full wave simulation result of an LWM prototype (untruncated in the lateral direction, obtained using COMSOL) designed to synthesize the same aperture. The two sets of results agree strongly, confirming the realizable focusing power of the q-BIC-based antenna.

Representative Work	Architecture	Capabilities and Key Advantages	Disadvantages
[11], [13]	Single-layered LWM with quasi-periodically modulated tensorial electric impedance	Arbitrary far-field beam-shaping with polarization and bandwidth control.	Requires multi-scale numerical optimization.<br><br>Cannot precisely control the near field.
[20],[21]	Single-layered LWM consisting of leaky waveguides loaded with tunable meta-atoms	Reconfigurable far-field radiation patterns.<br><br>Simple design framework facilitated by sparsely spaced meta-atoms.	No control over phase and polarization.<br><br>Limited angular range and radiation efficiency.<br><br>Cannot precisely control the near field.
[6], [10]	Multi-layered LWM with locally engineered electric and magnetic susceptibilities	Complete near-field and far-field control with high precision.	Requires multi-layered meta-atoms which are difficult to design and implement.
This work	Single-layered LWM supporting q-BIC	Complete and arbitrary control of near-field and far-field shaping of polarization, wavefront shape, with four degrees of freedom per unit cell, within a simple, rationally designed analytical framework.	Possible limitations over the angular range and overall radiation efficiency.

Supplementary Table S1: Comparison of the proposed LWM with selected prior art.
